# Modeling the interplay between structural plasticity and spike-timing-dependent plasticity

**DOI:** 10.1186/1471-2202-16-S1-P107

**Published:** 2015-12-18

**Authors:** Richard M George, Peter U Diehl, Matthew Cook, Christian Mayr, Giacomo Indiveri

**Affiliations:** 1 Institute of Neuroinformatics, University Zurich, Zurich, Switzerland; 2 ETH Zurich, Zurich, Switzerland

## 

Structural Plasticity describes a form of long-term plasticity, in which the pruning and the creation of synapses lead to the formation of memories in the topology of a network of neurons. In contrast, classical learning rules such as spike-timing dependent plasticity (STDP) focus on changing the efficacy of synapses, for example by looking at the correlation of pre-and post-synaptic activity in spiking neural networks. Typically, prolonged correlated activity leads to a long-term potentiation of the synaptic weight, while anti-correlated activity depresses the weight.

**Figure 1:**
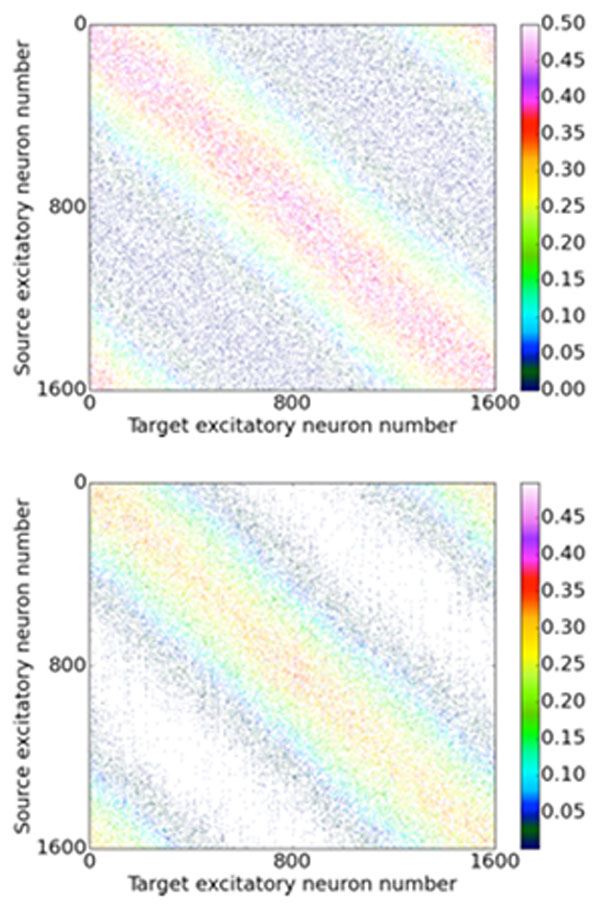
**Connectivity matrix from input to target neuron population with and without structural plasticity**. Top: Randomly initialized connectivity, learned using triplet STDP. Bottom: Connectivity learned using structural plasticity and triplet-STDP.

We propose a computational model that combines classical learning rules with structural changes in spiking neural network architectures that are based on observations on the morphological changes real biological synapses undergo during their live-cycle. Our model is based on the assumption that newly formed synapses are initially silent, due to their lack of AMPA receptors. In these synapses, only co-activation with other synapses can lead to postsynaptic potentials, and if this co-activation is not present for a critical period, the synapse degenerates again [1]. To study the interaction of structural plasticity and classical STDP learning rules, we simulated a highly recurrently connected spiking neural network and presented topological inputs to its neurons. We implemented the triplet STDP learning rule proposed by Pfister and Gerstner [2], and applied a structural plasticity rule where a critical period is opened whenever a synaptic weight is decreased below a certain threshold. If the weight does not manage to reach a set threshold by the end of the critical period, the synapse is pruned, and a new synapse is instantiated within the network; otherwise the synapse is maintained. This approach implies a homeostasis in the number of consolidated synapses in the network, while keeping the connectivity at a desired level of sparseness. We show in Figure 1 simulation results in which the input topology of the network is first learned using only STDP, and then, after activating structural plasticity, the structure of the connectivity matrix itself is adapted such that it reflects the input topology.

A major advantage of structural plasticity in artificial neural networks is given by the fact that it allows a drastic increase in performance given a finite number of synaptic resources. In addition to offering a promising approach for optimizing performance in software simulated networks, the model we propose optimizes the usage of resources in dedicated hardware neural network implementations that are faced with limited resources for emulating or simulating synaptic connections. This is particularly relevant for electronic implementations of spiking neural networks, ranging from GPU-based systems to mixed signal analog-digital neuromorphic VLSI devices.
